# System Identification Methodology of a Gas Turbine Based on Artificial Recurrent Neural Networks

**DOI:** 10.3390/s23042231

**Published:** 2023-02-16

**Authors:** Rubén Aquize, Armando Cajahuaringa, José Machuca, David Mauricio, Juan M. Mauricio Villanueva

**Affiliations:** 1 Universidad Nacional de Ingeniería, Rimac 150101, Peru; 2 Universidad Nacional Mayor de San Marcos, Lima 15081, Peru; 3 Universidade Federal da Paraíba Campus I, Joao Pessoa, Paraíba 58051-900, PB, Brazil

**Keywords:** method, gas turbine, neural network, NARX, identification system, prediction performance

## Abstract

The application of identification techniques using artificial intelligence to the gas turbine (GT), whose nonlinear dynamic behavior is difficult to describe through differential equations and the laws of physics, has begun to gain importance for a little more than a decade. NARX (Nonlinear autoregressive network with exogenous inputs) is one of the models used to identify GT because it provides good results. However, existing studies need to show a systematic method to generate robust NARX models that can identify a GT with satisfactory accuracy. In this sense, a systematic method is proposed to design NARX models for identifying a GT, which consists of nine precise steps that go from identifying GT variables to obtaining the optimized NARX model. To validate the method, it was applied to a case study of a 215 MW SIEMENS TG, model SGT6-5000F, using a set of 2305 real-time series data records, obtaining a NARX model with an MSE of 1.945 × 10^−5^, RMSE of 0.4411% and a MAPE of 0.0643.

## 1. Introduction

For the most part, power production plants use fossil fuels or nuclear energy. However, with the depletion of fossil fuels and environmental pollution problems due to greenhouse gas emissions, the proportion of power generated from renewable energy has gradually increased to replace other power generation systems [1]. The production of energy from those that are renewable varies according to natural conditions, such as wind speed and solar radiation. For this reason, it has been studied how to overcome the problem of the intermittency of renewable energies and their storage [2]. Faced with this problem, attention is being paid to the Gas Turbine (GT), which has the fastest response among conventional power generators, which is convenient so as not to affect the stability of electrical networks [3].

A GT has a high specific power and emits much fewer pollutants because it uses natural gas as fuel. In addition, it can achieve fast starts and stops compared to other power generation systems, such as coal and nuclear power generation, and fast load-following operation. Recently developed GTs use the combustion of hydrogen and natural gas mixtures, making greener operations possible [4].

For reasonable control and monitoring of the operation of the GT, a model is required that represents with excellent approximation its real dynamic behavior. There are two approaches to modeling a GT: the white box and the black box.

White box models. The white box models of the GT describe its behavior using physical equations based on engineering principles and its dynamics. These models are used when there is sufficient knowledge about the physics of the system [5], and they can be nonlinear [6,7] or linearized [8,9].

Several investigations have been carried out in the field of identification of the behavior of a GT using white-box models, among them, for adaptive control [10], for a low-power TG [11], for a heavy-duty power plant [12], for a micro GT [13], for a GT of a power plant based on the Rowen model [14], for a robust control system of a GT, etc.

Nonlinear GT models are usually in the form of large simulation codes and many details. Due to their complexity, they are not generally used for controller design and system stability analysis. On the other hand, the linearized models only consider the input and output variables, so the intermediate variables are not included in the model, losing quality for the control of the GT [15].

Although it is true that, to model a GT, physical equations that govern the real dynamics of the TG can be used with excellent approximation, these are pretty complex. Then, there will always be inaccuracies due to the unmodeled dynamics and parametric uncertainties [15]. In addition, detailed information on its components is needed to develop a physical model, and usually, this information is kept reserved for manufacturers and limited to outsiders.

Black box models. Its objective is to model the behavior of a GT for a set of input data $\left(u_{1},u_{2},\cdots ,u_{n}\right)$ and output $\left(y_{1},y_{2},\cdots ,y_{n}\right)$ when little or no data is available on information about the physical system [16,17]. One of the most important methods in this type of modeling is the artificial neural network (ANN) since they have demonstrated high precision in industrial applications [6], good operation and control systems [18], and, in addition, allow the optimization of the fuel consumption of the GT [19].

A type of ANN that has shown excellent results is the Nonlinear AutoRegressive network with eXogenous inputs (NARX) [20] because it contemplates feedback of the output variable and delays in the input variables. NARX applications for GT with a layer are found in turboshaft engines [5], aircraft engines [21], and in improving starting operation [22]. An application of NARX with two layers for modeling a GT is found in [17].

Studies show NARX models for specific cases of a GT, whose efficiency depends on the number of layers, number of nodes in each layer, input variables, number of delays, and data. However, no studies have been identified that present procedures to systematically design a NARX for any case of a GT, this being the objective of this paper.

The main contributions of this article are: (a) Provide a general review of the NARX black box models for identifying a GT, its concept, and modeling. (b) Provide a systematic method for designing a NARX model to identify a GT. (c) Show the method’s usability through its application to a real case of electric power generation through a heavy-duty single-axis GT.

This article is organized into six sections, and Section 2 reviews the operation of the GT. Section 3 reviews the NARX model applied to a GT. Section 4 proposes the systematic method for the design of the NARX model. Section 5 describes a case study to test the proposed method. Finally, in Section 6, the conclusions are presented.

## 2. Operation of the GT

The GT is an internal combustion engine that uses the air’s gaseous energy to convert the fuel’s chemical energy into mechanical energy and works according to the Brayton cycle [23].

**Figure 1:**
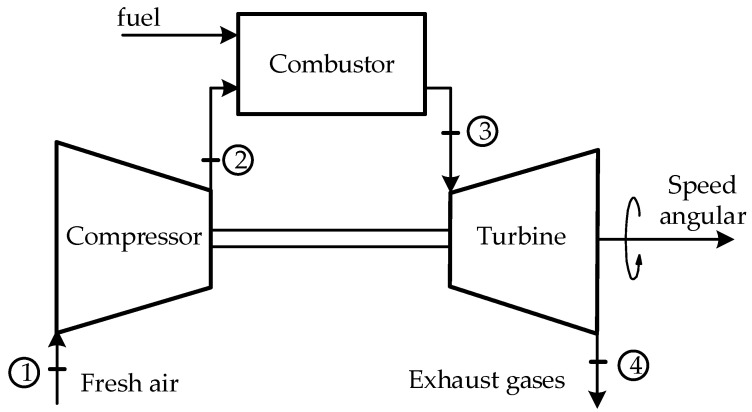
Typical schematic of an open-loop GT system.

Figure 1 shows a typical schematic of a GT system made up of an air compressor, a combustor, and a turbine. Air at atmospheric pressure enters the compressor (1), which is increased by the compressor at its outlet (2), and enters the combustion chamber to mix with the fuel and is ignited to produce hot expanding gas that enters (3) and drives the turbine to generate mechanical energy on its axis that rotates at a certain angular speed, in order to drive electric generators, pumps, compressors, among others. Finally, the gases leave the turbine (4).

The ideal Brayton cycle consists of two isobaric and two isentropic processes [24]. The two isobaric processes consist of the combustion system of the gas turbine and the gas side of the HRSG (Heat Recovery Steam Generating). The two isentropic processes represent compression and expansion processes.

**Figure 2:**
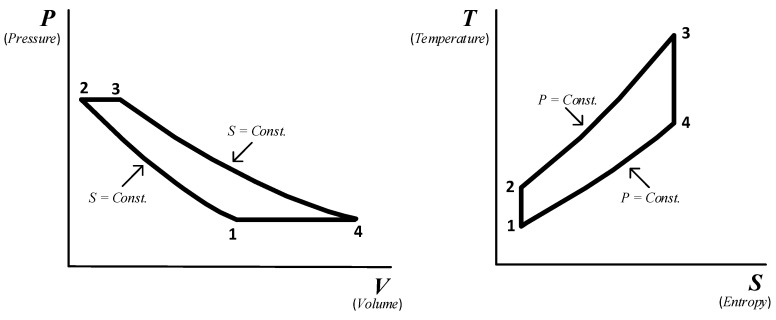
The air-standard Brayton cycle.

Figure 2 presents a simplified application of the first law of thermodynamics to the Brayton cycle of standard air in the absence of changes in kinetic and potential energy.

The expressions for compressor work $\left(W_{c}\right)$, Turbine work $\left(W_{t}\right)$, total output work $\left(W_{cyc}\right)$, heat added to the system $\left(Q_{2,3}\right)$ and efficiency total cycle $\left(\eta_{2,3}\right)$, are formulated with Equations (1) to (5), respectively [7].

(1) $$W_{c}={\dot{m}}_{a}\left(h_{2}-h_{1}\right)$$

(2) $$W_{t}=\left({\dot{m}}_{a}+{\dot{m}}_{f}\right)\left(h_{3}-h_{4}\right)$$

(3) $$W_{cyc}=W_{t}-W_{c}$$

(4) $$Q_{2,3}={\dot{m}}_{f}xLHV_{fuel}=\left({\dot{m}}_{a}+{\dot{m}}_{f}\right)-{\dot{m}}_{a}h_{2}$$

(5) $$\eta_{2,3}=W_{cyc}/Q_{2,3}$$

where ${\dot{m}}_{a}$ is the air mass, ${\dot{m}}_{f}$ is the mass of fuel, $LHV_{fuel}$ is the lower heating of fuel and $h_{1},$ $h_{2}$, $h_{3}$, $h_{4}$ are the enthalpies of states 1 through 4, respectively.

The higher the turbine ignition pressure-temperature ratio, the greater the efficiency of the Brayton cycle. The total cycle efficiency relationship is based on the simplifying assumptions: (1) ${\dot{m}}_{a}\gg {\dot{m}}_{f}$, (2) the gas is calorically and thermally perfect, that is, the specific heat at constant pressure ($c_{p}$) and the specific heat at constant volume ($c_{v}$) are constant, and the ratio of specific heat *γ* remains constant throughout the cycle, (3) the pressure ratio (*r_p_*) of the compressor and the turbine are the same, and (4) components work at 100% efficiency. Under these assumptions, the efficiency of the ideal Brayton cycle, operating between room temperature and ignition temperature, is given by Equation (6):

(6) $$\eta_{ideal}=\left(1-\frac{1}{r_{P}^{\frac{\gamma -1}{\gamma }}}\right)$$

where $r_{P}\;$= Pressure ratio; and *γ* is the ratio of the specific heats.

Supposing that $r_{P}$ is the same in the compressor and the turbine, the following relationships hold.

(7) $$\eta_{ideal}=1-\frac{T_{1}}{T_{2}}$$

with $r_{P}$ in the turbine.

(8) $$\eta_{ideal}=1-\frac{T_{4}}{T_{3}}$$

In a real cycle, taking into account the efficiencies $\eta_{c}$ of the turbine compressor and the efficiency $\eta_{t}$ of the expander, the efficiency of the entire cycle between the ignition temperature $T_{f}$ and room temperature $T_{amb}$ of the turbine is given by Equation (9).

(9) $${}_{cycle}=\left(\frac{\eta_{t}T_{f}-\frac{T_{amb}r_{p}^{\left(\frac{\gamma -1}{\gamma }\right)}}{\eta_{c}}}{T_{f}-T_{amb}-T_{amb}\left(\frac{r_{p}^{\left(\frac{\gamma -1}{\gamma }\right)}-1}{\eta_{c}}\right)}\right)\left(1-\frac{1}{r_{p}^{\left(\frac{\gamma -1}{\gamma }\right)}}\right)$$

The maximum t“I have checked and revised all”otal efficiency of the thermal cycle, considering the optimal pressure ratio for fixed inlet temperatures and efficiencies for the compressor and the turbine, is obtained with the following relationship.

(10) $${\left(r_{p}\right)}_{copt}={\left\{\frac{1}{T_{1}T_{3}\eta_{1}-T_{1}T_{3}+T_{1}^{2}}\left[T_{1}T_{3}\eta_{t}-\sqrt{{\left(T_{1}T_{3}\eta_{t}\right)}^{2}-\left(T_{1}T_{3}\eta_{t}-T_{1}T_{3}+T_{1}^{2}\right)\left(T_{3}^{2}\eta_{c}\eta_{t}-T_{1}T_{3}\eta_{c}\eta_{t}+T_{1}T_{3}\eta_{t}\right)}\right]\right\}}^{\frac{\gamma -1}{\gamma }}$$

So that there are no losses in the compressor and the turbine, in Equation (10), it must be considered *η_c_* = *η_t_* = 1, with which the optimal pressure ratio is reduced to:

(11) $${\left(r_{p}\right)}_{copt}={\left(\frac{T_{1}T_{3}}{T_{1}^{2}}\right)}^{\frac{\gamma }{\gamma -1}}$$

The optimum pressure ratio for the maximum output work of a turbine, taking into account the efficiencies of the compressor and the turbine expander, is given by Equation (12).

(12) $$r_{p_{wopt}}={\left[\left(\frac{T_{3}\eta_{c}\eta_{t}}{2T_{1}}\right)+\frac{1}{2}\right]}^{\frac{\gamma }{\gamma -1}}$$

## 3. The NARX Model

The NARX model is a type of ANN with feedback suitable for non-linear modeling systems, especially time series, that use past measurements to predict future values [25].

**Figure 3:**
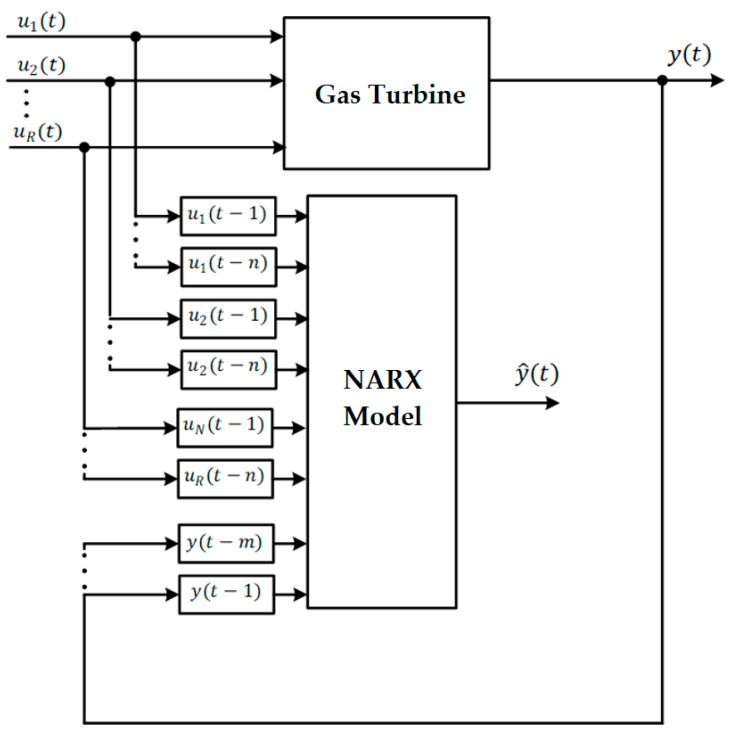
Structure of the NARX Serial-Parallel model for the GT.

In Figure 3, the NARX model is illustrated, where the “System” represents a real or artificial process to be approximated, and the “Model” represents the system that allows its behavior to be simulated. NARX can be used in series-parallel mode or parallel mode. The series-parallel mode predicts one or more future steps based on past exogenous inputs (*u*) and outputs (*y*) of the system. On the contrary, in parallel mode, the model’s output is considered instead of the output of the system [17]. Considering that the model output may have an error, these could negatively influence the model outputs in relation to the system, so in this work, we will consider the series-parallel mode.

(13) $$U\left(N,n\right)=(U_{1}\left(n\right),\;\ldots ,U_{R}\;\left(n\right))$$

(14) $$U_{i}\left(n\right)=(u_{i}\left(t-1\right),\ldots u_{i}\left(t-n\right)),\;\forall i=1,\ldots ,n$$

The future value of the output signal $\hat{y}\left(t\right)$ depends on its m past values $Y\left(t,m\right)$ and on R×n past exogenous inputs $U\left(R,n\right)$. The function *f* approximates the behavior of the TG. NARX can be implemented using a feedforward neural network to approximate the function f [26].

## 4. Systematic Method to Build the NARX Model

**Figure 4:**
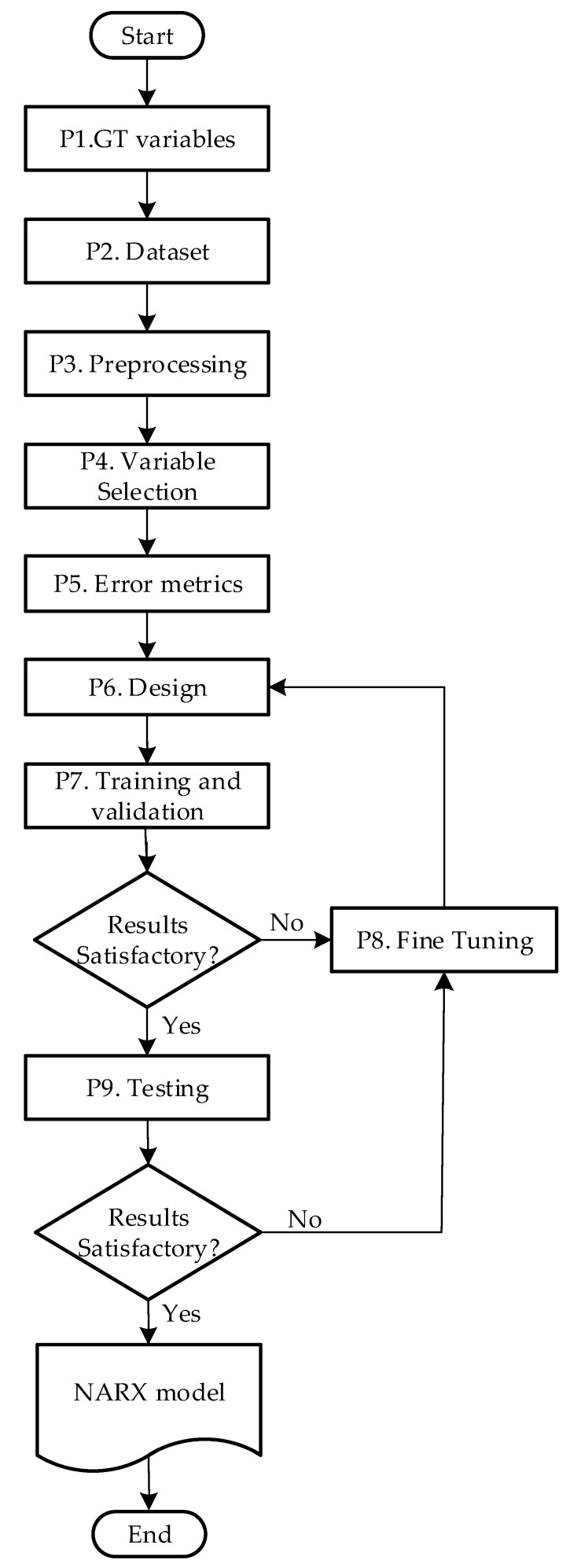
Method for the systematic design of NARX.

A method for the systematic design of a NARX model with high precision for a GT is proposed, based on nine steps (Figure 4), which go from identifying variables to obtaining a GT’s identification model. Each of the steps is described below:

### 4.1. GT Variables (P1)

The first step in any design of a model for a GT is the definition of its input and output variables. The output variables refer to the behavior of the GT to be identified, such as the angular velocity [27], the outlet temperature [20], the output power, and the thermal efficiency [28]. The input variables, also called exogenous, are the variables that influence the behavior of the GT, such as the compressor inlet temperature [10], the compressor inlet pressure, the fuel flow, and the Inlet Guide. Vane (IGV) [22]. All these variables allow for knowing the dynamic behavior of a GT. Depending on the TG to be identified, the input and output variables of the GT must be identified.

### 4.2. Dataset (P2)

In this step, a dataset is determined on the input and output variables identified in step *P1*. The dataset can be obtained by simulation [29] or measuring the GT under study [30]. In either case, it is required that data cover one operating cycle of the GT, that the period between data recordings is constant and as small as a few minutes or seconds, and that data are reliable. The dataset, in turn, will be divided into three datasets, one for training, another for validation, and one for testing, with some authors considering proportions of 70–15–15% [16] or 60–15–25% [31], where a–b–c% are the proportions of the dataset for training (a%), validation (b%) and testing (c%).

### 4.3. Preprocessing (P3)

This process aims to place the dataset ready for processing, that is, to be used in the training, validation, and testing process. For this, the following activities [32,33] are carried out if the case warrants: integration of data in various repositories, cleaning, imputation, and normalization.

### 4.4. Variable Selection (P4)

The purpose of this step is to select the input variables that are correlated with the output variable. In this way, we can understand the behavior of the GT. For this purpose, there are various techniques, such as the Principal Component Analysis evaluator [34,35] and the Pearson correlation [36], some of them implemented in libraries such as Python, Pytorch, Keras, and in tools such as MatLab and Waikato Environment for Knowledge Analysis (Weka).

### 4.5. Error Metrics (P5)

The error metrics that are commonly used to evaluate and report the performance of a regression model are, among others: the mean absolute error (MAE) [28], the mean square error (MSE) [37,38], the error mean absolute percentage (MAPE) [39], and root mean square error (RMSE) [40]. Let $\hat{y}\left(t\right)$ be the output of the model, $y\left(t\right)$ the output of the GT, and N the number of records to evaluate, then these metrics are determined as:

(15) $$MAE=\frac{1}{N}\overset{t=N}{\underset{t=1}{\sum }}\left|y\left(t\right)-\hat{y}\left(t\right)\right|$$

(16) $$MSE=\frac{1}{N}\overset{N}{\underset{t=1}{\sum }}{\left(y\left(t\right)-\hat{y}\left(t\right)\right)}^{2}$$

(17) $$MAPE=\frac{1}{N}\overset{N}{\underset{t=1}{\sum }}\left|\frac{y\left(t\right)-\hat{y}\left(t\right)}{y\left(t\right)}\right|$$

(18) $$RMSE=\sqrt{\frac{\sum_{t=1}^{N}{\left(y\left(t\right)-\hat{y}\left(t\right)\right)}^{2}}{N}}$$

### 4.6. Design (P6)

The design of a NARX implies a network structure that includes all the elements for it to learn to identify a GT [41,42]. For what should be considered: input variables, output variables, an input layer, and an output layer, one or more hidden layers, number of neurons in each hidden layer, transfer functions, such as logsig, purelin, hardlim, satlin, and poslin, activation function, propagation function, training function, such as trainlm and trainbfg, and number of delays of the output signal and input signals. In addition, values for the hyperparameters, the number of epochs, the learning rate, the momentum rate, and the desired final error must be considered.

Some tools allow configuring a NARX topology quickly, such as MATLAB and Python, in their current versions.

### 4.7. Training and Validation (P7)

In this step, the NARX model designed in step 6 is trained using the training dataset and learning algorithms, such as backpropagation [43,44] and Q-Learning [45]. The model was validated based on cross-validation techniques. These algorithms were implemented in MATLAB [46,47], Weka [44,48], and Python, as well as its frameworks, such as Tensor Flow and Keras [49].

### 4.8. Fine Tuning (P8)

This step is applied only if the model obtained in step 7 presents unsatisfactory results (metrics).

Fine-tuning is the procedure that aims to determine which hyperparameter values (number of layers, number of nodes per layer, types of activation functions for each layer, initial weights, etc.) provide the model with better results in the learning process [50]. In general, fine-tuning is implemented in various tools that provide machine learning algorithms, some fine-tuning code being GridSearch or RandomizedSearch.

### 4.9. Testing (P9)

In this step, the successful NARX model obtained in step 7 is tested against the validation dataset, and the error metrics given in step 5 are measured. If the results of this step are satisfactory, that is, an acceptable error, then an adequate NARX model is obtained, and the process is complete; otherwise, it must return to step 6 to build a satisfactory new model. For testing, the same tools are used for training.

Satisfactory NARX model. The final model will be obtained from step 9 if the result of the metrics used is satisfactory. In this way, the model will allow the representation of a GT’s behavior with the desired accuracy.

Next, a case study is developed to illustrate the application of the proposed method.

## 5. Case Study: Single Shaft Open Cycle Gas Turbine

**Figure 5:**
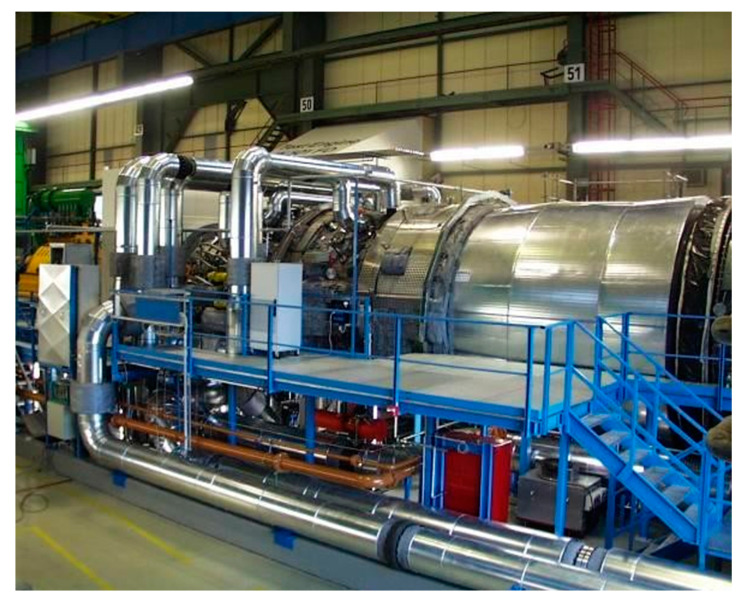
A view of the SIEMENS brand gas turbine, model SGT6-5000F [51].

The GT of Lima (GTL) is from 2009 and is a single-axle, heavy-duty, whose characteristics and a view of it are shown in Table 1 and Figure 5, respectively. It works five days a week, 24 h a day, Monday through Friday, and is used by a private electricity generation company in Peru. In the present case study, the GTL output variable of interest is the rotational speed of its axis.

**Figure 6:**
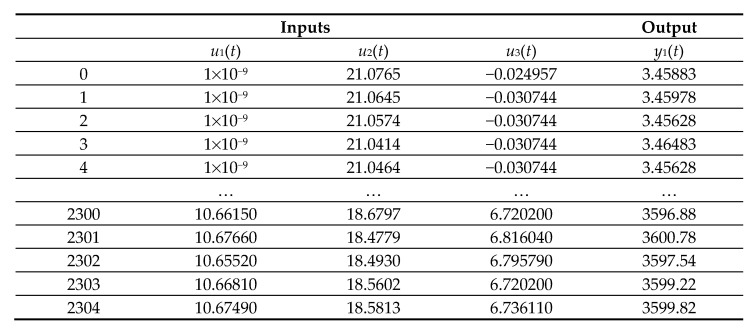
A sample of the dataset.

Next, the application of the proposed method step by step for the GTL is shown. The input and output variables of the GTL are shown in Table 2. Data were obtained through transmitters of all the variables, through a data logging system of the control and supervision system SPPA-T3000 control system of SIEMENS, during eight days, every 5 min, considering the cycle of intermittent starts and stops, obtaining 2305 records in Excel, which are available upon request. Figure 6 shows a sample of the dataset. It has been considered to divide the dataset for training, validation, and testing into 70%, 15%, and 15%, respectively.

Since data were correctly obtained by the SCADA data logging system, no integration, cleaning, or data imputation was required. However, to avoid biases of one variable, data were normalized, proportionally passing all data to a scale from 0 to 1. The variation of the values of the variables $u_{1}$, $u_{2}$(*t*), $u_{3}$(*t*), $u_{4}$(*t*), $u_{5}$(*t*) and $y_{1}$(*t*) before normalization is from 10^−9^ to 10.879533 kg/s, from 14.758274 to 21.932644 °C, from −0.0604022 to 5.960286 INH_2_O, −2.258088 at 198.91522 MW, 98.76779 at 99.30859 kPa, and 3.4272156 at 3634.8547 RPM, respectively.

**Figure 7:**
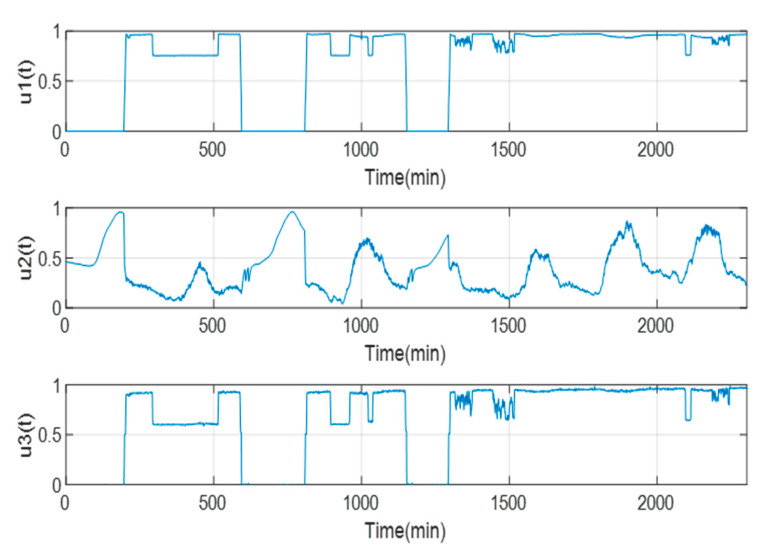
Behavior of the normalized data of the input variables and the output data of the GTL.

For the selection of the input and output variables, the InfoGain AttributeEval program of the Weka version 3.4.8 tool was applied, through which the input variables $u_{4}\left(t\right)$ and $u_{5}\left(t\right)$ were discarded. Thus, the selected variables are $u_{1}\left(t\right)$, $u_{2}\left(t\right)$ and $u_{3}\left(t\right)$. In addition, to confirm the selected variables, the Pearson correlation coefficient was used, which shows a value greater than 0.95. Figure 7 shows part of the behavior of the normalized data for the three input variables.

**Figure 8:**
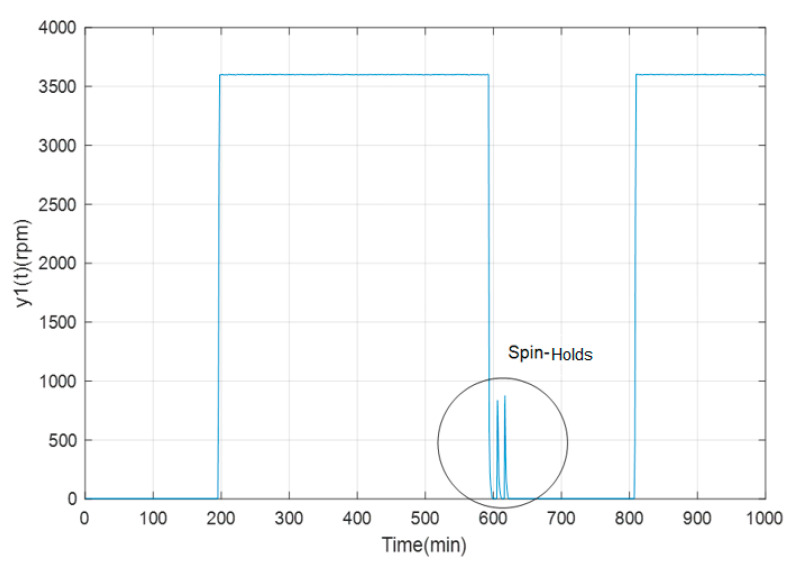
Spin-Holds of the angular velocity of the TGL.

Figure 8 shows part of the spin-holds of the angular velocity, which constitute short periods of rotation of the GTL, driven by a prime motor and not by gas, with the purpose of its cooling. Therefore, when it keeps rotating, the fins of the GTL produce cooling. These spin-holds occur between the time intervals of 600–650 min and 1150–1200 min.

The metrics used to evaluate the performance of the NARX models are MSE and MAPE, whose calculations are given in Equations (4) and (5), respectively, and where $\hat{y}\left(t\right)$ is the model output and N is the number of records of the dataset to be evaluated.

For the design of the NARX model of the TGL, the MATLAB tool (v. 20–22a) was used with the three selected input variables and the output variable; likewise, one or more hidden layers were considered with a variation of neurons per layer, plus various backpropagation training functions, transfer functions, and one or more lags on the input and output variables. On the other hand, the number of epochs was set equal to 100, the learning rate at 0.01, the momentum rate at 0.9, and the desired error of no more than 1%.

Next, the NARX model established in the design is trained using the training dataset. An iterative process in two loops has been considered to determine the values of the hyperparameters that provide the best results for the model. The first is to tune the hyperparameters in the training and validation process (Step P7), and the second for the testing process (Step P9). In each loop, the number of neurons has been varied from 1 to 30 for each hidden layer. Tests have been carried out with different backpropagation training functions and a combination of other transfer functions for the hidden and output layers. In addition, 1 to 4 delays have been considered.

The NARX model, obtained by the training and validation processes and fine-tuning, is tested with the Testing dataset to know the model’s performance through the metrics (Step P9). Steps P6–P9 were repeated until a model was obtained that provided satisfactory results in the validation process.

**Figure 9:**
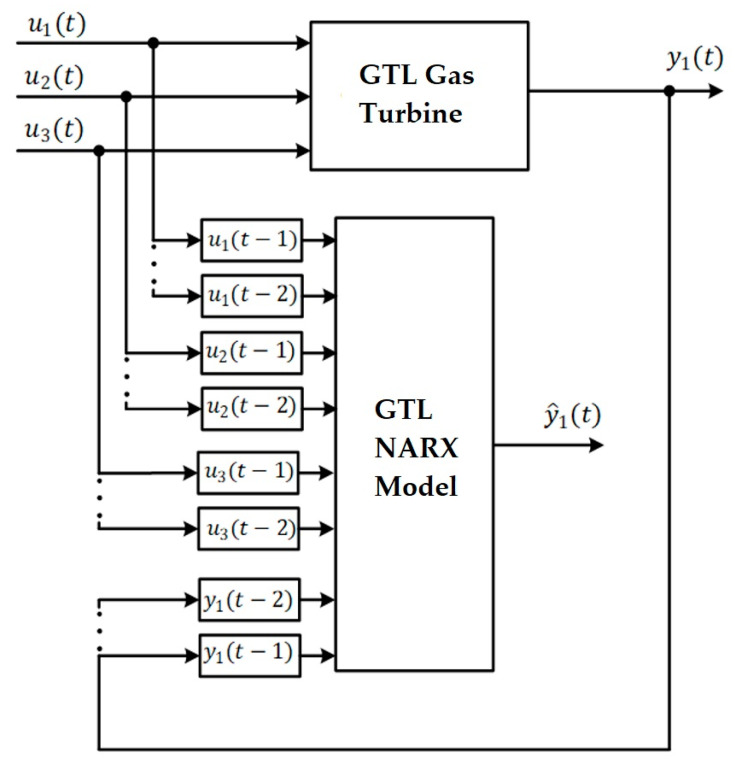
NARX model for the GTL.

After carrying out all the experiments, the NARX model was finally obtained (Figure 9), which we will call optimal NARX, with the hyperparameters: Number of input and output delays = 2; Number of hidden layers = 2, Number of neurons in the first layer = 15, Number of neurons in the second layer = 3; Transfer function = Trainlm; Activation function = Logsig with a value between 0 and 1; Number of epochs = 100; Desired final error = 10^−4^; Learning rate = 0.01; momentum rate = 0.9; Minimum Relative Absolute Error = 1%.

**Figure 10:**
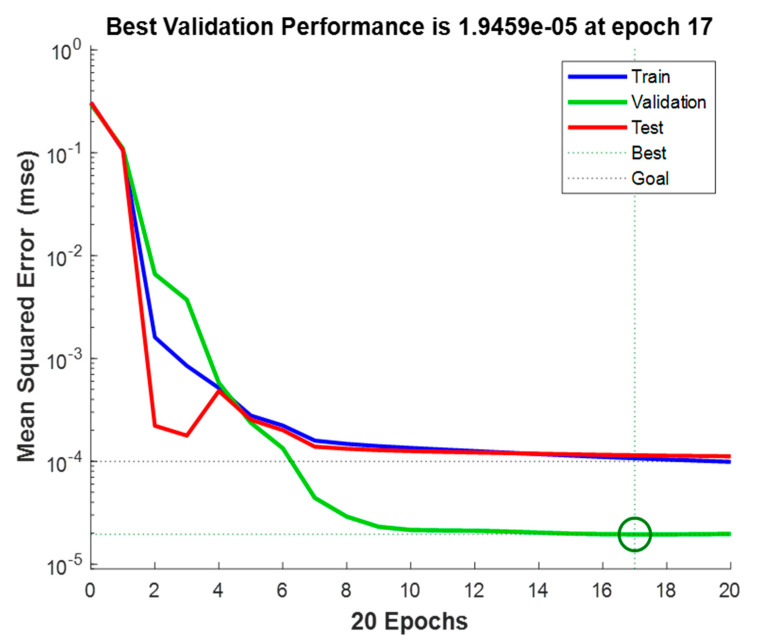
Optimal NARX network performance.

Figure 10 indicates the performance of the optimal NARX model (obtained with the proposed method), reaching an MSE of 1.9459 × 10^−5^ in epoch 17, an RMSE of 0.4411%, and a MAPE of 0.0643.

**Figure 11:**
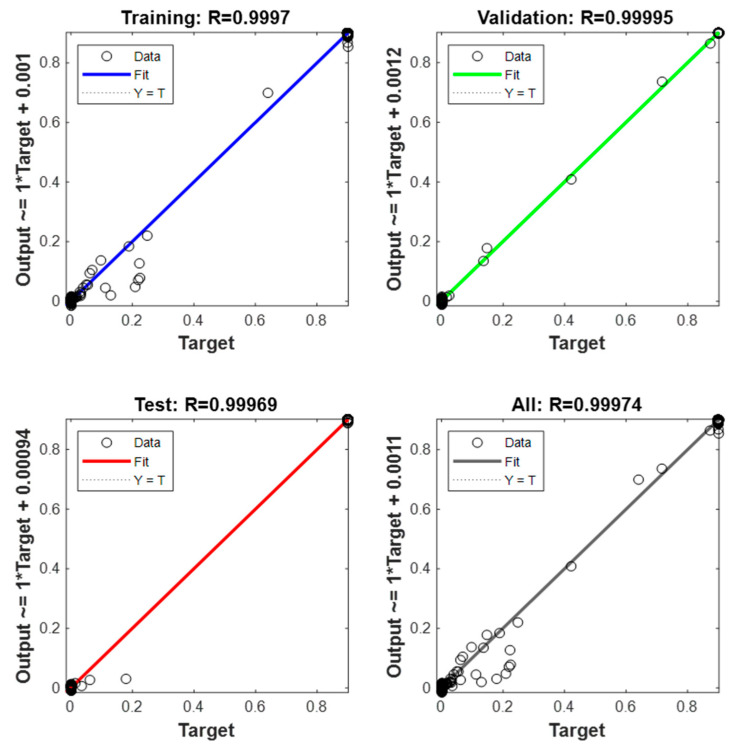
Regression of the optimal NARX network.

**Figure 12:**
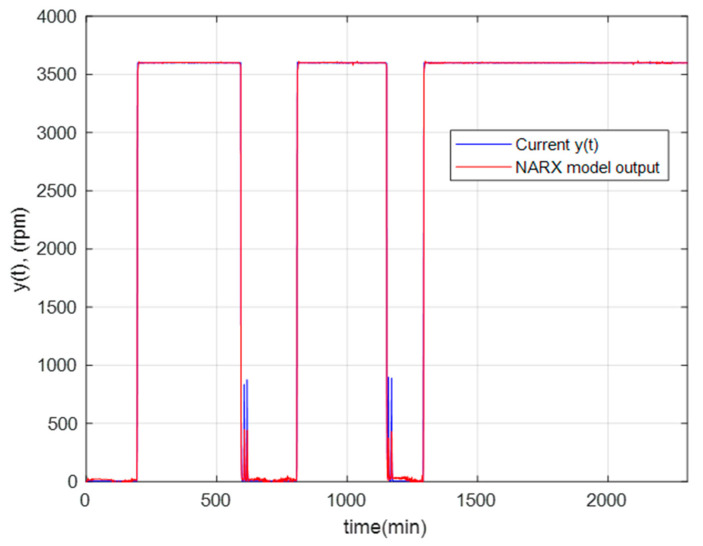
Behavior of the real angular velocity and the optimal NARX model for the GTL.

Figure 11 shows the regression graph, which indicates the relationship between the NARX network’s output and the system’s output (objective). The R-value is an indication of the relationship between outputs and goals. As the figure shows, the R values for all plots are very close to 1. Therefore, the result for each training, validation, and testing data set indicates a good fit. Figure 12 shows the behavior of the real angular velocity (blue) and that obtained by the optimal NARX model (red) for the GTL.

**Figure 13:**
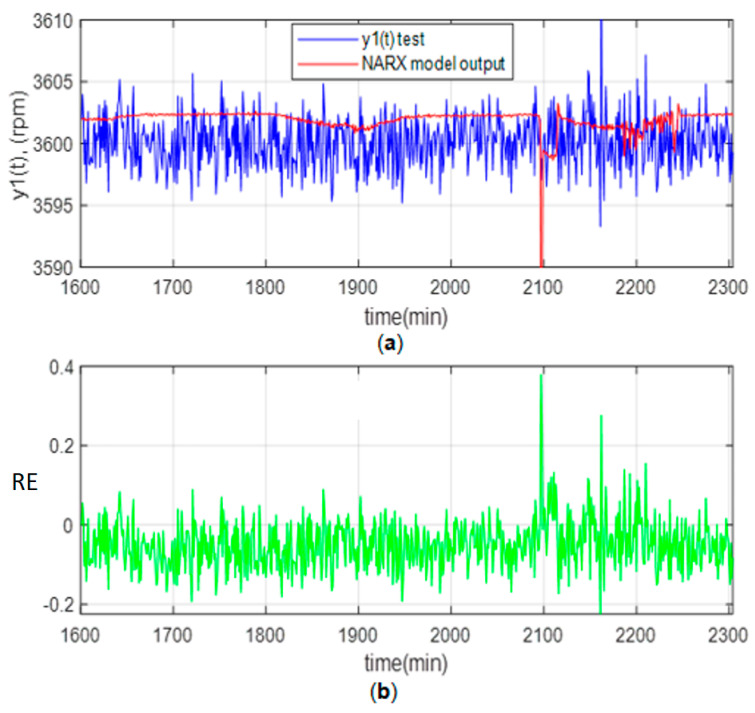
Test results: (**a**) Angular velocity; (**b**) Relative Error (RE).

Figure 13a shows the angular velocity variations for the Testing and the optimal NARX between 3595 and 3605 rpm. It should be noted that the expected angular speed is 3600 rpm. Figure 13a shows that between 2100 and 2250 min, a transient occurs in the turbine load, which was controlled automatically. Likewise, Figure 13b shows the relative error (RE). 

## 6. Conclusions

This article proposes a systematic method for designing and developing black box models based on NARX neural networks to identify a robust, accurate, and reliable GT model. The proposal consists of 9 well-defined steps that go from identifying GT variables to obtaining a satisfactory NARX model.

In order to validate the proposed method, a case study was carried out using a dataset of 2305 records obtained by measuring the input and output variables of a SIEMENS brand gas turbine, model SGT6-5000F of 215 MW, located in Peru, called GTL. A computer program code was generated and executed in the MATLAB environment (v. 20–22a), following the proposed method step by step, systematically achieving a NARX model for the GTL. The training, validation, and testing results of the generated NARX model did not present significant deviations between the simulated and measured data. The values of the MSE, RMSE, and MAPE metrics during the training, validation, and testing phases were satisfactory, less than 10^−5^; 0.5% and 0.06, respectively, values that are very competitive compared to other similar cases.

The case study shows that the proposed method for the development of a NARX model is adequate to identify a GT and to predict with high precision its output parameters based on the changes in the system inputs. Furthermore, since the method is indifferent to the type of gas turbine, it can be applied to predict the behavior of similar gas turbine systems with high accuracy.

This applied research work has as future activities the development of advanced control systems using artificial intelligence techniques. In addition, new neural network architectures can be considered, such as LSTM.

## Figures and Tables

**Table 1:** GTL Specifications (SIEMENS, SGT6-5000F).

Characteristic	Value
Number of axes	1
Rotational speed	3600 rpm
Compression ratio	15.8
Inlet temperature	599 °C
Outlet temperature	1327 °C
Airflow range	571 kg/s
Power	215 MW
Heat ratio	9643 kJ/kWh
Efficiency	39.5 %

**Table 2:** GTL input and output variables.

Input Variables	Output Variables
*u*_1_(*t*): Gas fuel flow (kg/s)	*y*_1_(*t*): Angular speed (RPM)
*u*_2_(*t*): Inlet air temperatura (°C)	
*u*_3_(*t*): Barometric pressure (INH_2_O)	
*u*_4_(*t*): Megawatt selected (MW)	
*u*_5_(*t*): Inlet pressure (kPa)	

## Data Availability

The private data presented in this study are available on request from the first author.
